# The Concept of WC-CrC-Ni Plasma-Sprayed Coating with the Addition of YSZ Nanopowder for Cylinder Liner Applications

**DOI:** 10.3390/ma16031199

**Published:** 2023-01-31

**Authors:** Marek Goral, Tadeusz Kubaszek, Wieslaw A. Grabon, Karol Grochalski, Marcin Drajewicz

**Affiliations:** 1 Research and Development Laboratory for Aerospace Materials, Rzeszow University of Technology, al. Powstancow Warszawy 12, 35-959 Rzeszow, Poland; 2 Faculty of Mechanical Engineering and Aeronautics, Rzeszow University of Technology, al. Powstancow Warszawy 12, 35-959 Rzeszow, Poland; 3 Institute of Mechanical Technology, Poznan University of Technology, M. Skłodowska-Curie Square 5, 60-965 Poznan, Poland

**Keywords:** plasma spray, cylinder liner, friction, YSZ, WC, CrC, coating

## Abstract

In the article, the new concept of plasma-sprayed coatings for the cylinder liner was presented. The new type of powder containing WC-CrC-Ni with a 5 and 10 wt. % addition of nano-YSZ powder was plasma-sprayed on aluminum 2017 alloy samples. The selection of optimal plasma-spraying parameters was made taking into account the thickness, porosity, and hardness of the coatings. For the coatings obtained according to the developed parameters, the analysis of their microstructure, chemical, and phase composition was performed. At the next stage, the friction coefficient of the developed coatings was tested and compared with the properties of a classic cast-iron cylinder liner. The obtained results suggest that the developed type of coating might be used for cylinder liner applications after a deeper friction analysis.

## 1. Introduction

For the production of coatings inside the cylinder liners, different methods have been developed. The oldest type of coating was developed in 1967 by the Mahle Company Ni-SiC coating, called ‘Nikasil’. Datta et al. [1] showed that a typical Ni/SiC electrodeposited layer has superior wear resistance compared to a cast-iron liner. This type of coating might be produced using a magnetic-field-assisted electrodeposition process [2]. Their tribological properties might increase with the addition of an ionic liquid to the lubricant [3]. Later on, other electrodeposited or electroless deposited coatings were developed. The use of electroless plating of the Ni-B-CeO_2_ coating also improves the wear properties of the aluminum cylinder liners [4]. Su et al. [5] proposed using plasma electrolytic oxidation for the production of a wear-resistant coating for cylinder liners. The electroplating of chromium on the cylinder liners increases the friction coefficient (COF) compared to the cast-iron cylinder [6]. The other concept of improving Al-Si cylinder liners is the electroplating of the MoS_2_ coating [7]. The DLC coating doped with silicon was considered for application in heavy-duty diesel engine cylinder liners [8]. Beriet et al. [9] developed the Fe-based nanocrystalline coating on cylinder liners using an electroplating process.

The CrN coating deposited in the PVD process was proposed by Singh et al. [10] for the improvement of hypereutectic Al-Si alloys used for cylinder liners. The mechanisms of wear of CrN and counterbody surfaces in this type of coating at higher temperature (in the range of 20–300 °C) are abrasive wear accompanied by slight oxidation

In fact, different thermal spray processes such as wire arc spray, plasma transfer arc, atmospheric plasma spray, as well as HVOF were applied to the internal area of the cylinder liners [11,12,13,14,15,16,17,18,19,20,21,22,23,24,25,26,27,28,29,30,31,32,33,34,35,36,37]. Kincarid and Witherspoon [11] developed the pulse Arc-Spray for the production of Al and Fe-based alloys. This technology was developed by the PSA group and optimized by Bolot et al. [12]. Rao et al. [13] proposed the use of plasma spraying of Fe–FeO–Fe_3_C as a replacement for cast-iron liners. Iron powders might be mixed with different oxides such as Al_2_O_3_ and ZrO_2_ in different compositions [14]. The addition of oxides increases the wear resistance of the coating compared to that of iron-based coatings. Cook et al. [15] from the Ford Company developed a cost-effective process of Plasma Transferred Wire Arc (PTWA) on the aluminum cylinder liner. They developed special carbon- and boron-doped steels [16]. Later, this technology was fully developed by the Daimler Chrysler company [17]. This type of technology was also applied by the PSA group. The developed steel coating produced by the PTWA process was characterized by lower friction compared to cast-iron cylinder liners at different temperatures [18]. In this process, the use of Fe coating characterized by higher hardness increases the wear resistance of cylinder liners [19]. The use of an internal diameter extension HVOF gun for coating production in cylinder bores was proposed by Byrnes et al. [20]. The delamination of the tribolayers produced by the HVOF process was the primary source of material removal during scuffing. This process was facilitated by the formation of cracks in FeAlO_3_ inclusions, as well as fractures along FeO veins between the iron splats [21]. The NiCr, Al_2_O_3_/TiO_2_, and Cr_3_C_2_/NiCr coatings produced by the combustion flame spray and the HVOF process were investigated by Biyiklioğlu and Tat [22]. The Oerlikon-Metco company developed its plasma-spray guns called Rotaplasma for the spraying of different materials inside cylinders [23] as well as different types of coating from the SUMEBore family [24]. Their use reduces fuel consumption by up to 2% [25]. Uozato et al. [26] developed a new type of Fe–C–Ni–Cr–Cu–V–B alloy for the Rotaplasma process on cylinder liners. They confirmed similar or better tribological and corrosion properties of the coating obtained compared to the cast-iron cylinder liner. Vencl et al. [27] showed that the main mechanism of the Fe-alloy plasma-sprayed coating is the delamination of splat structures and the detachment of all parts of the coating, most of all the large oxides and precipitates. The addition of Cr_2_O_3_ to the Fe-based plasma-sprayed coating for the production of cylinder liner coatings was investigated by Liu et al. [28]. Song [29] analyzed the influence of an alumina and zirconia oxide addition on the wear resistance of Fe-based plasma-sprayed coatings. He observed that the coating with the oxide addition changes the main wear mode from the abrasive to the delamination one, in which cracked oxide/matrix interfaces fall off from the worn surface. Lee et al. [30] proposed the addition of Mo to the Fe plasma-sprayed coating. He observed that the optimal addition of molybdenum is 10% and it increases the mechanical properties in comparison with cast-iron liners. The higher concentration of Mo in the multicomponent plasma-sprayed coating for the cylinder liner was investigated by Pandey et. al. [31]. Mao et al. [32] showed that the Al_2_O_3_-13 wt. % TiO_2_ plasma-sprayed composite coating on the cylinder liner with an additional laser re-melting process is characterized by better mechanical properties. The use of a self-fluxing plasma-sprayed coating NiCrBSi after heat treatment was proposed by Liu et al. [33]. He showed that heat treatment improves the hardness of the coating but does not influence the friction coefficient. The influence of the plasma-spraying parameters of the NiCrBSi coating on its properties was analyzed by Tang et al. [34]. He proved that flow of primary plasma gas has a strong influence on wear resistance as well as on the friction coefficient. Wang et al. [35] showed that the plasma spraying of nickel on aluminum cylinder liners decreased the friction coefficient without the necessity of a honing process. The surface finish by honing thermally sprayed coatings was investigated by Hoffmeister et al. [36]. The casting requirements for thermally sprayed coatings on cylinder lines were analyzed by Ernst et al. [37] using microscopy methods. The most important was the base material/coating interface area.

The literature review indicates that various processes and materials are currently being used to enhance the properties of coatings applied to cylinder liners, particularly to improve abrasion resistance and hardness. A relatively new approach is the use of nanopowder additives to commercially available powders to improve specific properties, depending on the nanopowder used [38,39]. Myalska et al. [38] investigated the effect of applying WC, Cr_3_C_2_ and TiC nanopowder additives to standard WC-Co powder at a weight ratio of 5–95. The addition of Cr_3_C_2_ and TiC nanopowders resulted in a decrease in coating porosity and fracture resistance, and an increase in coating microhardness. According to our previous work [40,41,42], we proposed the addition of nano-YSZ powder to the fine-grain WC-NiCr powder and its atmospheric plasma spraying (APS) on an aluminum base material to increase the wear resistance and reduce the coefficient of friction of the cylinder liner. As the mentioned studies show [38], the addition of 5 wt. % nanopowder can significantly improve the properties of thermal-sprayed coatings. According to the potential application of the proposed cylinder liner coatings, the high nano-YSZ powder content in the carbide powder might decrease the thermal conductivity of the coating. For this reason, the thermal conditions of the engine operation may be disturbed. Therefore, two small additive contents of nano-YSZ powder (5 and 10 wt%) to conventional carbide powder were used in the presented study.

## 2. Materials and Methods

### 2.1. Plasma-Spraying Process and Coating Properties Analysis

The aluminum alloy 2017 type was used as a base material in the form of 5 mm metal sheet. Atmospheric plasma spraying (APS) was conducted using A60 plasma-spraying system (Thermico, Dortmund, Germany). The powder for thermal spraying was prepared using Thermico SJA 175 base material with the following composition (wt. %): WC-73%, CrC-20%, and Ni-7%. Metco 6609 powder (yttria-stabilized zirconia oxide, YSZ) was used as an additive (5 and 10 wt. %) to SJA 175 powder typically used in Suspension Plasma Spraying (SPS) method. The powders were mixed using ball-milling process for 2 h. The pure SJA 175 powder was also plasma sprayed using parameters developed in our previous research [42]. The experimental processes included a change in the power current, composition of plasma gases, and powder feed rate (Table 1). The feed speed of the torch was 50 mm/min, and the spraying distance was 100 mm. The selection of spraying parameters was conducted for powder containing 10 wt. % of Metco 6609 YSZ.

After coating deposition, metallographic samples were prepared for each set of spraying parameters. The microstructure analysis of developed coatings was conducted using a Phenom XL Scanning Electron Microscope (Thermo Fischer Scientific, Waltham, MA, USA) equipped with an EDS spectrometer. The thickness and porosity were calculated using the NIS Elements software (Nikon, Japan). For XRD, hardness and friction measurements the samples were designated in Tables 2–4 and Figures 8–12 as:

A pure cast iron (grade EN-GJL-250) cylinder liner;
B sample with optimal parameters plasma-sprayed SJA 175 powder;
C sample with optimal parameters plasma-sprayed SJA 175 + 5 wt. % of YSZ addition;
D sample with optimal parameters plasma-sprayed SJA 175 + 10 wt. % of YSZ addition.

The XRD phase analysis was conducted using an X-ray diffractometer ARL X’TRA (CuKα radiation Bragg–Brentano geometry value of the angle 20–90°, Thermo Scientific Corporation, Waltham, MA, USA). For the identification of the phase components, the ICDD-PDF4-2019 crystallographic database was used. The hardness of coatings was measured using Nexus 4303 (Innovates, Maastricht, The Netherlands). The used load was 4903 N. The measurement time was 10 s. A grey cast-iron sample hardness was also measured. As the materials are heterogeneous, the hardness measurement was performed using the Brinell method and the result was converted to the Vickers scale for comparison with the hardness of the deposited coatings. The Brinell method was measured using a ZHU 250 hardness tester (ZwickRoell, Ulm, Germany). The ball diameter was 2.5 mm and the load was 18,387.47 N. For each sample, the average was calculated from 5 measurements.

**Figure 1:**
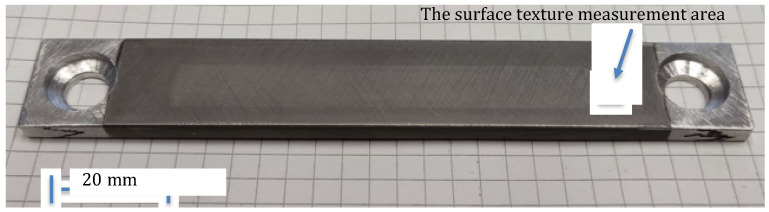
Example of B-type sample, with the ticking of the surface texture measurement area.

For friction measurement, the special type of samples with coatings produced using optimal parameters were used. The typical example of the B-type sample is shown in Figure 1, and the area where the surface texture is measured is also visible. Counter-specimens were prepared on the base of piston rings made of grey cast iron. These elements were characterized by a flat ring-running profile.

Measurement of the surface topography of the samples near the return position of the ring was made using the Talyscan 150 (Taylor Hobson, Warrenville, IL, USA) measuring device with the TalyMap Expert software. The measurement speed was 2 mm/s and the vertical resolution was about 15 nm. The measuring area was 1.5 mm × 1.5 mm. The sampling interval in the measurement direction and in the direction perpendicular to it was 5 μm. The form was removed by a third-degree polynomial. In Table 2, there are the values of selected surface topography parameters.

### 2.2. Method of Friction Resistance Analysis

**Figure 2:**
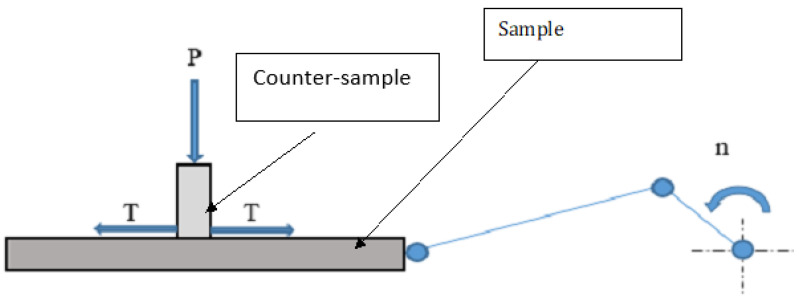
Schematic representation of the tribological tester, P—load, T—friction force, n—rotational speed converted to reciprocating motion.

To perform friction resistance research, a reciprocating motion tribological tester was used. The general principle of operation of the tribological tester is shown in Figure 2.

The radius of the crank is 45 mm, giving a frictional length of 90 mm. To regulate the speed, a frequency converter that co-operated with a TAMEL three-phase induction motor by TAMEL was used. It is possible to adjust the engine speed in the range of 0 to 2850 rpm.

The tribological research was performed to answer whether the developed coatings will reduce resistance to motion. Each of the friction pairs was subjected to a tribological test consisting of two basic stages: running-in and basic tests. Before starting the running in, the samples were evenly covered with 2 mL of oil (SAE viscosity grade: 0W–30). During the running-in process, the average sliding speed was 0.18 m/s, while the load of the sample with the normal force (P) was changed every 2 min, starting from 50 N to 300 N (the increase was 50 N). The total working time of the friction pairs in the running-in stage was 12 min. After the running-in period, new oil was introduced in the amount of 2 mL. The oil was evenly distributed throughout the sample.

The main tests were carried out for three different reciprocating frequencies:

1. 5 Hz—At this reciprocating frequency, the working time of the friction assembly was 90 min;
2. 7.55 Hz—At this reciprocating frequency, the working time was 88 min;
3. 10 Hz—At this reciprocating frequency, the working time was 2 min.

The load of the sample during the main tests was constant and amounted to 300 N. Two replications were carried out for each type of sample. The wear of the specimens was measured gravimetrically via the change in weight. For this purpose, the specimens were weighed before and after the test with an ABT-1204M (Kern, Balingen, Germany) balance with a repeatability of 0.1 mg.

## 3. Results and Discussion

### 3.1. Influence of Plasma-Spraying Parameters on Properties of Coatings

**Figure 3:**
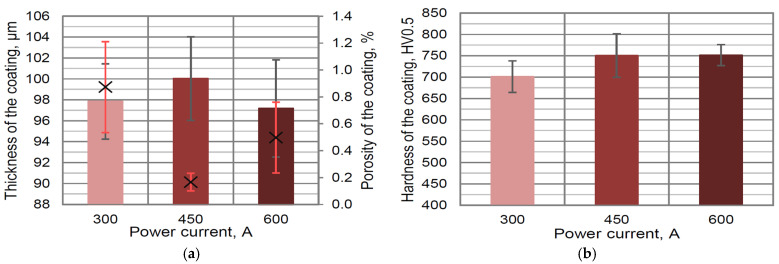
The influence of power current on thickness/porosity (in the figure, the porosity is marked as x) (**a**) and hardness (**b**) of developed coating containing 10 wt. % YSZ additions.

The thermal spraying of the newly developed powder was conducted using different values of the following parameters: power current, plasma gasses composition, and powder feed rate. The relationship between coating thickness and power current showed that decreasing the current value to 300 A does not allow for full particle melting, and as a result, the thickness on the level of 98 μm as well as the highest porosity was obtained (approx. 0.8%) (Figure 3a). When a medium value of power current (450 A) was used, the thickness above 100 μm and the lowest porosity (0.16 vol. %) were measured. The coating formed using the highest power current was characterized by porosity on the level of approx. 0.5 vol. %; additionally, the lowest thickness (below 98 μm) for all power currents was obtained (Figure 3a). This might be connected with the high energy of the plasma plume and the decomposition of some carbide particles during deposition. The observed porosity was much lower in comparison with previously analyzed pure Thermico SJA 175 powder [42] and was similar to those obtained using HVAF processes [38]. The average hardness of the coating increased to about 750 HV0.5 with the increasing of the power current up to 450 and 600 A (Figure 3b).

**Figure 4:**
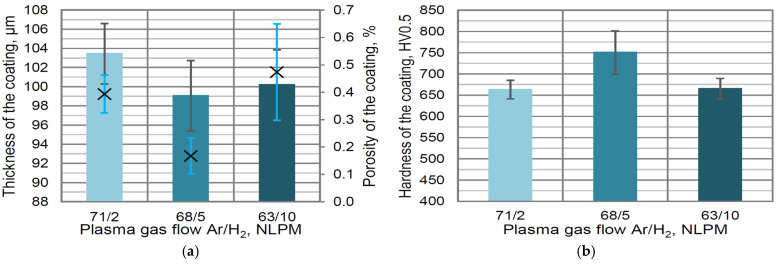
The influence of plasma gas composition on thickness/porosity (in the figure, the mean value of the porosity is marked as ×) (**a**) and hardness (**b**) of developed coating containing 10 wt. % YSZ additions.

The influence of the plasma gas composition (especially the hydrogen flow rate) was a second analyzed factor for plasma-spraying parameter selection. The use of a low flow rate of hydrogen in a plasma plume (2 NLPM) does not enable full particle melting and finally provides a high porosity of the obtained coating (0.39 vol. %) (Figure 4a). A similar effect was observed when the high concentration of hydrogen (10 NLPM) increases the plasma plume energy and, due to the partial decomposition of carbides, forms a higher porosity (about 0.5 vol. %). The thickness of the coatings obtained was in the range of 99–103 μm (Figure 4a). The highest hardness—750 HV0.5—was measured when the medium value of hydrogen flow (68/5 NLPM) rate was used (Figure 4b). The lower hardness (663 and 665 HV0.5) was measured for two values of the H_2_ flow rate, (71/2 NLPM) and (63/10), respectively (Figure 4b).

**Figure 5:**
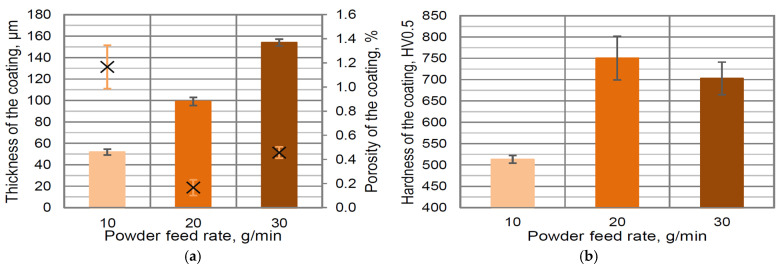
The influence of power feed rate on thickness/porosity (in the figure, the mean value of the porosity is marked as ×) (**a**) and hardness (**b**) of developed coating containing 10 wt. % YSZ additions.

The linear trend of increasing coating thickness according to increasing powder feed rate was measured (Figure 5a). It is a typical relationship for all thermal spray materials and methods [43]. The large deviation in the porosity value was measured. The highest porosity of coatings formed using a low powder feed rate (10 g/min) has no explanation and requires further research (Figure 5a). For this value of feed rate, the lowest hardness (513 HV0.5) was measured (Figure 5b). For comparison, the increasing of the powder feed rate to 30 g/min enables us to obtain a coating characterized by higher hardness—approx. 703 HV0.5.

The results showed that a medium set of parameters (I = 450 A, H_2_ flow 5 NLPM, powder feed rate 20 g/min) allows us to obtain the coatings with an optimal combination of the thickness (99 μm), porosity (0.16 vol. %), and hardness (751 HV0.5). These values of parameters should be used for the preparation of coatings. The achieved coatings were characterized by porosity similar to those obtained using high-velocity thermal spray processes such as HVOF [44], HVAF [39], and cold spray [45].

### 3.2. Microstructure, Phase Composition, and Hardness of Obtained Coatings

**Figure 6:**
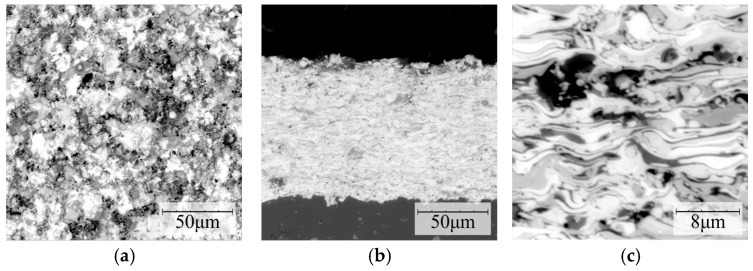
Surface morphology (**a**) and microstructure (**b**,**c**) of developed coating with the addition of 10 wt. % of YSZ plasma-sprayed using optimal parameters (I = 450 A, H_2_ flow 5 NLPM, powder feed rate 20 g/min).

The developed coating with the addition of 10 wt. % of YSZ was characterized by a surface morphology typical for plasma-sprayed carbide coatings [46] (Figure 6a). A large number of splats and particles were observed (Figure 6b). Cracks in the coating were not observed. The typical lamellar structure of the sprayed coatings was observed with the presence of some pores and oxides (Figure 6c).

**Figure 7:**
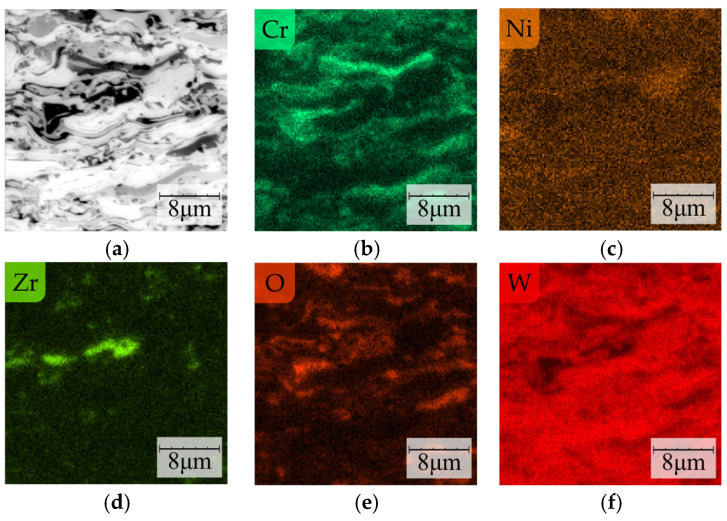
The cross-section of selected area in developed coating (**a**) and elemental mapping of Cr (**b**), Ni (**c**), Zr (**d**), O (**e**), and W (**f**).

For structure identification, elemental mapping by the EDS method was used (Figure 7a). Nickel was observed in the whole analyzed area and formed a metal matrix of metaloceramic coating (Figure 7c). In contrast, chromium was detected only in selected areas—it was connected with the formation of chromium carbides (Figure 7b). Tungsten was present in most of the areas analyzed except for areas where increased levels of zirconium and oxygen were found (Figure 7d–f). In areas rich in Zr and O, the added YSZ powder was likely to be present. The results of oxygen mapping also suggest the partial oxidation of the metallic components of the coating during plasma spraying (Figure 7d,e).

**Figure 8:**
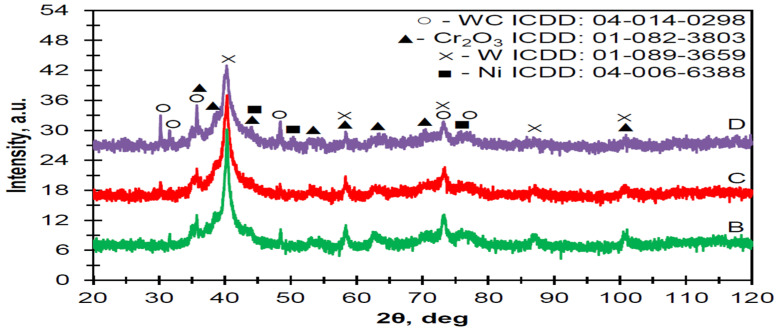
The XRD pattern of pure plasma-sprayed Thermico SJA 175 powder (B) with addition of 5 wt. % (C) and 10 wt. % (D) of YSZ powder.

The phase composition was identified for three types of coatings: pure carbide metaloceramic powder (Thermico SJA 175) and two concentrations of additional YSZ powder: 5 and 10 wt. % (Figure 8). The obtained results showed the presence of the main components of the basic powder: tungsten and chromium carbides as well as pure nickel and tungsten. The presence of the YSZ additive was not identified on the XRD spectra. It might be connected with the low value of the added yttria-stabilized zirconia, which was not sufficient to be identified. The presence of Cr_2_O_3_ on the spectra suggests the partial oxidation of chromium during the plasma-spraying process.

**Figure 9:**
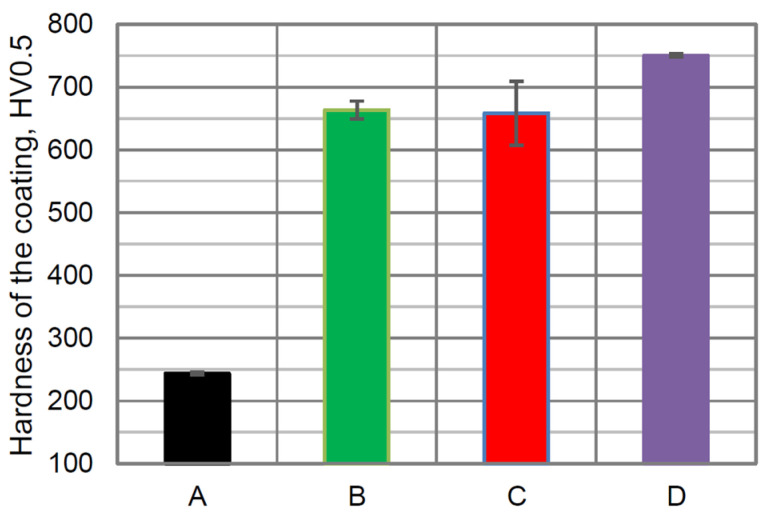
The hardness of pure cast-iron cylinder liner (A) and plasma-sprayed coatings using: pure SJA 175 powder (B) with 5 wt. % (C) and 10 wt. % (D) addition of YSZ powder.

The measured hardness of typical cast-iron cylinder lines was very low—243 HV0.5 (Figure 9, sample A). For comparison, the hardness of pure SJA 175 (B) and with the addition of 5 wt. %. of YSZ powder (Figure 9, sample C) increased more than twice to about 650 HV0.5. When the concentration of the YSZ powder addition increased to 10 wt. %, the hardness was much higher, approx. 750 HV0.5 (Figure 9, sample D). It might be concluded that this value of the YSZ nanopowder addition enables an increase in the hardness up to 100 HV0.5 in comparison with pure SJA 175 powder. During thermal spraying, the decarburization process of WC and CrC takes place when high plasma energy is used [47,48]. The YSZ was not decomposed during plasma spraying and as a result, the higher hardness of sample D was measured.

### 3.3. Friction Processes

**Figure 10:**
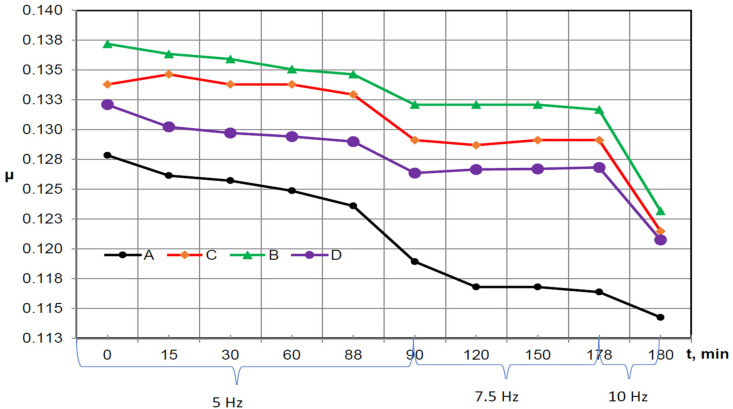
Visualization of changes in the coefficient of friction with time for individual friction pairs with constant load and variable reciprocating frequency (Samples: cast iron (A), pure SJA 175 powder (B) and with 5 wt. % (C) and 10 wt. % (D) addition of YSZ powder.

Changes in the friction coefficient over time for individual types of samples at different reciprocating frequencies are shown in Figure 10. The same as in [49], the friction coefficient was calculated as the ratio of friction force (T) to normal force (P). The stabilized maximum values of the friction coefficient for the total time of 2 min. measured in different periods are shown in this figure.

The characteristic of the results presented in Figure 10 is that the friction coefficient has the highest value at the beginning of the test and then it starts to decrease while the tests are in progress. This is related to the continuous running-in of the tested friction pairs especially during the first 90 min when the reciprocating frequency was on the level of 5 Hz. Analyzing the friction coefficient from the point of view of the reciprocating frequency, it can be seen that in the 90th minute of the test, after increasing the reciprocating frequency to 7.5 Hz, there was a decrease in the mean values of the friction coefficient. A similar situation can be seen in the 178th minute when the reciprocating frequency was increased to 10 Hz.

This observation remains in agreement with the result presented in [50,51] where the increase in the reciprocating frequency causes a decrease of COF, whilst in [50], the increase in the frequency from 2 to 4 Hz causes a decrease of the COF for smoother types of surfaces in contrast with the results were obtained for surfaces characterized by greater roughness; however, it should be highlighted that these tests were realized with a load of 6 N and the temperature 150 °C.

Table 3 presents the relative scatters of friction force for various reciprocating frequencies and normal loads. Similarly to [49], the scatter between the two test replications is defined as the difference in the ratio of the friction force to its average value. The average range of friction force dispersion for the combination with the A-type sample is 2.97%, and the friction force spread for the C-type samples is similar (2.83%) (Table 3).

The most stable operation conditions were provided by the B-type samples, for which the average friction force spread was 2.2%. Despite the good results in terms of the mean value of the friction coefficient obtained by the D-type samples, they were burdened with the highest dispersion of the friction force, which was at the level of 6.4%.

Direct observation of the tests shows that the lubricant remained in the friction zone of the sample and counter-sample until the end of the test. In the case of friction assemblies containing sample types B, C, and D, a significant share of wear products was found in the lubricant. These observations were confirmed by a weight analysis of the counter-samples, including a comparison of the weights of the counter-samples before and after the tribological test (Table 4). The results of these analyses indicate that in the case of counter-samples co-operating with samples of type B, C, and D, there was a weight loss at the level of 2.3%, 1.56%, and 1.36%, respectively. However, for counter-samples co-operating with type A, this loss was at the level of 0.1%. It should be noted that after the completion of the tribological tests, no weight loss was recorded in any of the sample sets.

**Figure 11:**
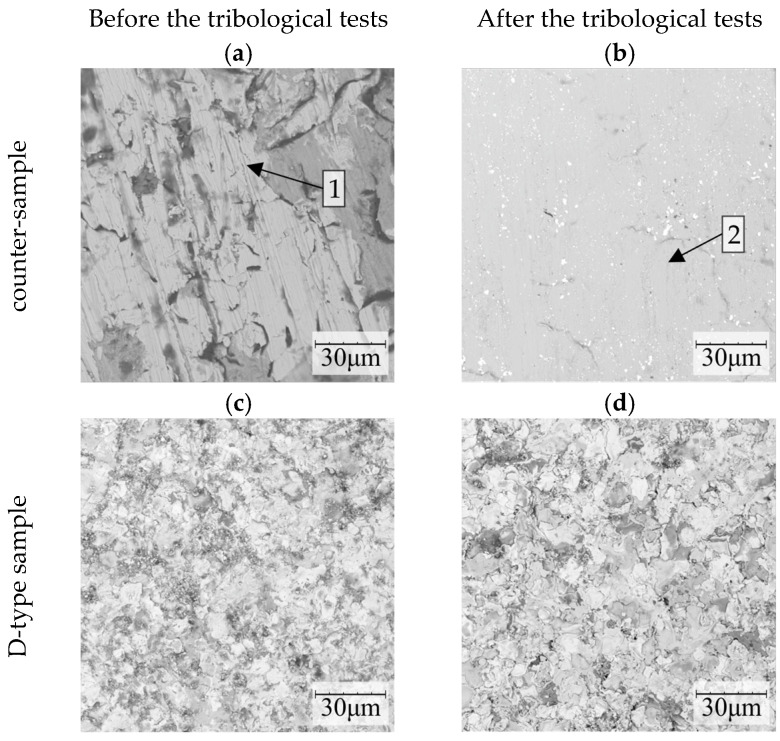
The surface morphology of the counter-sample (**a**,**b**) that co-operated with the D-type sample (**c**,**d**) with the deposited carbide coating with the addition of 10 wt%. YSZ, before and after the tribological tests. 1—scratches being an effect of the treatment process, 2—scratches being an effect of the wear process.

A detailed SEM analysis of the surface morphology before and after the tribological tests, shown in Figure 11, allows us to confirm that the piston ring (counter-sample) was worn in the tested friction pairs. This is in accordance with the above-described change in the mass of the samples after the test. The surface of the counter-sample has been smoothed and only small scratches are visible in the direction of reciprocating motion realized during the test (Figure 11b). Regardless of the type of specimen with the deposited carbide coating (B, C and D), no signs of wear were observed on the surface of the coating after the tribological test (Figure 11c,d).

**Figure 12:**
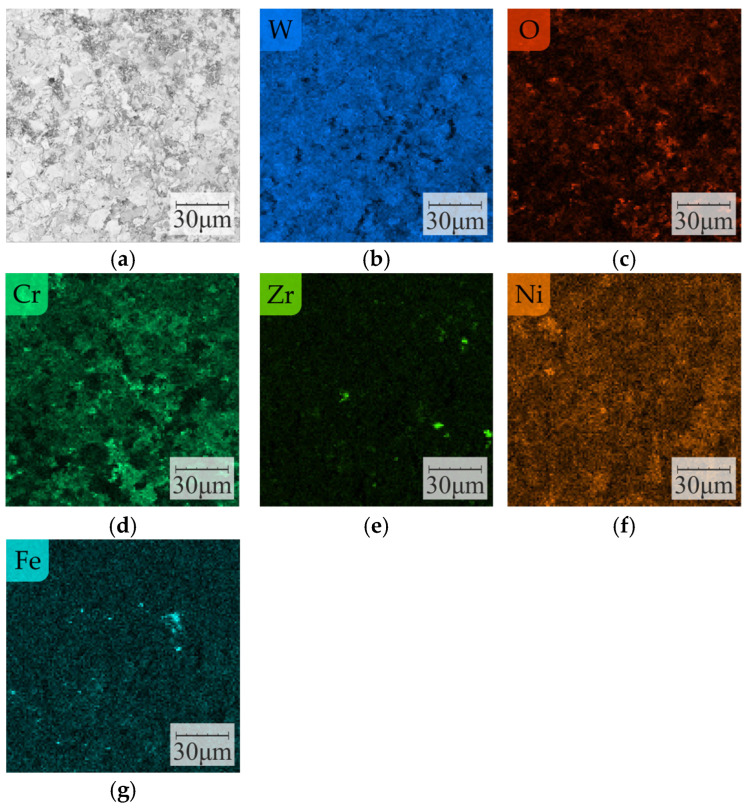
The surface morphology of the D-type sample (10 wt. % addition of YSZ powder) after the tribological test (**a**) and elemental mapping of W (**b**), O (**c**), Cr (**d**), Zr (**e**), Ni (**f**), and Fe (**g**).

Analysis of the chemical composition of the surface of the samples after the test (Figure 12) showed the presence of elements of carbide powder used in the deposition of the coatings, that is, W, Ni, and Cr, as well as Zr particles from the added YSZ nanopowder (Figure 12b,d–f). Visible particles of O on the surface indicate oxidation (probably Cr) during the plasma-spraying process of the coating (Figure 12c). Significantly, Fe particles were observed on the surface of the carbide-coated samples regardless of their type (B, C, and D), originating from the abrasion of the counter-sample in the tribological test, confirming the transfer of material from the counter-sample to the sample during friction in the tribological test (Figure 12g). On the other hand, no coating-derived elements were observed on the surface of the counter-sample after the test.

Based on this information, it should be highlighted that the standard material used to produce the piston rings has insufficient properties to co-operate with the coatings used to produce samples B, C, and D; therefore, other materials or additional coatings such as molybdenum [52] or other multicomponent coatings [53,54] could be used and verified in future research.

In the group of samples containing the newly developed coatings, the D-type samples obtained the best results. The analyses show that the results obtained are related to the fact that these samples were characterized by the highest hardness among the samples with newly introduced coatings. It was connected with the higher hardness of coating achieved by the addition of 10 wt. % of YSZ powder (approx. 750 HV0.5) in comparison with original WC-CrC-Ni powder (about 650 HV0.5). These samples also had the lowest values of the surface texture Sa parameter. The results obtained are related to the fact that these samples were characterized by the value of the Spk parameter at the level of 0.21 µm, and therefore had the lowest mean height of peaks above the core surface among all the samples tested, which had a direct impact on the contact mechanics and thus on the COF values. The values of the Svk parameter of this type of surface indicate that the valley section allows for retaining the oil, permitting the formation of an appropriate thickness of the oil film, and allowing the reduction of the COF in a friction pair containing such samples. It should also be emphasized that the values of the mean square surface gradient described by the Sdq parameter, which was at the level of 0.067 (in the case of a perfectly flat surface, the value of this parameter is equal to 0), also contributed to lower friction.

However, it should be noted that in the period from 90 to 178 min. for the reciprocating frequency on the level of 7.5 Hz, especially for samples C and D, the coefficient of friction tends to increase probably due to the influence of the accumulated wear debris in the oil, which is directly connected with the fact that these samples were characterized by the lowest values of the Svk parameters, which have a direct impact on the lower possibility of accumulation of the wear debris in the valley section of these samples compared to the A and B types of samples.

The C-type samples were unable to achieve results as good as the D-type samples. This was because, despite similar valley roughness, they had about 33% higher mean height of peaks above the core surface characterized by the Spk parameter. The Sa, Sk, and Sdq parameters of these samples were also higher compared to those of the D-type samples.

The average value of the coefficient of friction up to 178 min of testing duration was the highest for B-type samples. Among the proposed solutions (B, C, and D), these samples had the highest value of the analyzed surface texture parameters, which probably translates into the result obtained. Relatively large valleys (described by the Svk parameter; it should be noted that the value of this parameter was the highest for the samples from groups B–D) of this type of samples probably influenced the good storage of wear products; this is evidenced by the fact that, during the tests, there was no increase in the friction coefficient in the assemblies containing this type of sample. The mentioned valleys undoubtedly also influenced the oil retention, and thus the good formation of the oil film allows us to obtain very low values of COF for the reciprocating frequency equal to 10 Hz.

It should be noted that cast iron, although characterized by high roughness, allowed one to achieve the lowest coefficients of friction. This is because the friction pairs containing these samples constantly undergo running-in during the tests (continuous decrease in the friction coefficient) and change the character from a one-process surface to a two-process surface characterized by the very smooth plateau region allowing the obtainment of lower values of COF; this phenomenon was detailed described in [50,51,55,56,57,58]. The parameters achieved also result from the physical properties of cast iron, in which carbon is in the form of graphite, which, due to its lubricating properties, helps form solid lubrication at the contact area [58], significantly reducing the friction coefficient.

Based on the results of the hardness and friction tests, it might be concluded that the using of a 10 wt. % YSZ addition to WC-CrC-Ni powder reduces the decarburization effect and increases the tribological properties and hardness of the coating [59]. The microstructural and geometrical measurement of the wear area formed during the friction test requires a deeper analysis using microscopic methods. In further research, the other wear tests for the developed coating such as cavitation [60,61] and ball-on disc [62] might be conducted.

## 4. Conclusions

- The new concept of WC-CrC-Ni powder modification was developed by adding nano-YSZ powder. The relationship between plasma-spraying parameters (power current, H_2_/Ar flow ratio, and powder feed ratio) and the thickness, porosity, and hardness of the coatings was analyzed.
- The reference parameters were selected as follows: power current I = 450 A, H_2_ flow 5 NLPM, and powder feed rate 20 g/min. The established values allowed us to achieve coatings with the optimal combination of the thickness (99 μm), porosity (0.16 vol. %), and hardness (751 HV05).
- The tribological properties of the WC-CrC-Ni plasma-sprayed coating with the addition of YSZ nanopowder for cylinder liner applications show that the D-type samples with optimal parameters plasma-sprayed SJA 175 + 10 wt. % of YSZ addition gives the lowest mean values of coefficient of friction; however, relative dispersion of the friction force, in this case, was the highest.
- The most stable operation conditions were provided by the B-type samples (sample with optimal parameters plasma-sprayed SJA 175 powder), for which the average friction force spread was 2.2%. However, for those samples, the mean values of the coefficient of friction were the highest. It should be emphasized that an increase in the hardness of the coatings resulted in a decrease in the COF.

## Figures and Tables

**Table 1:** The variable and constant plasma-spraying parameters used in experimental.

Parameter	Value
Power current, A	300, 450, 600
Hydrogen/Argon flow rate, NLPM (total flow 73 NLPM)	2/71, 5/68, 10/63
Powder feed rate, g/min	10, 20, 30
Carrier gas flow rate, NLPM	6
Spray distance, mm	100
Injection nozzle diameter, mm	1.8
Plasma gun feed rate, mm/s	1000
Time of deposition, s	180

**Table 2:** Mean values of selected surface texture parameters of particular types of samples (A–D).

Sample Designation *	Sa, µm	Sk, µm	Spk, µm	Svk, µm	Sdq
A	0.94	2.41	1.44	2.23	0.11
B	0.70	2.05	0.41	1.36	0.094
C	0.50	1.45	0.28	1.01	0.079
D	0.39	1.02	0.21	0.98	0.067

* Sample A–D composition described in the text of Section 2.1.

**Table 3:** Relative dispersion of the friction force [%].

Reciprocating Frequency	5 Hz					7.5 Hz				10 Hz
Sample Type *	Time [min]									
	0	15	30	60	88	90	120	150	178	180
A	0.66	2.02	2.70	2.72	3.44	4.29	3.64	5.09	4.38	0.74
B	3.10	1.87	1.25	0.00	0.63	1.93	1.93	1.93	2.58	6.90
C	4.44	5.68	4.44	4.44	1.92	1.32	0.66	1.32	2.63	1.40
D	7.72	6.78	8.25	5.45	6.12	4,37	5.23	5.83	5.63	9.00

* Sample A–D composition described in the text of Section 2.1.

**Table 4:** A weight loss of counter-samples co-operating with samples of type A, B, C, and D.

Counter-Samples Co-Operating with Samples *	Average Weight before Tribological Test, g	Average Weight after Tribological Test, g	Average Relative Weight Loss, %
A	0.9391	0.9382	0.10
B	1.1170	1.0913	2.30
C	1.1355	1.1179	1.56
D	1.1291	1.1137	1.36

* Sample A–D composition described in the text of Section 2.1.

## Data Availability

Not applicable.
